# Exploring the role of circular RNAs in endometrial cancer progression and chemoresistance: Implications for targeted therapies

**DOI:** 10.1186/s40001-025-03401-w

**Published:** 2025-11-26

**Authors:** Xin Wen, Yanhong Zhai, Gaoli Niu, Hong Wang, Luhong Men, Yajuan Ren, Xuejiao Xing, Lingli Zhao, Nannan Liu

**Affiliations:** Department of Gynecologic Oncology, The First Affiliated Hospital of Henan Polytechnic University (The Second People’s Hospital of Jiaozuo), Jiaozuo, 454000 China

**Keywords:** Circular RNA, Endometrial cancer, Chemoresistance, Targeting mechanism, Programmed cell death

## Abstract

Endometrial cancer stands as a prevalent malignant tumor of the female genital tract, while showing growing incidence statistics. Circular RNAs (circRNAs) demonstrate translational value as biomarkers and therapeutic targets in endometrial cancer because they affect chemoresistance and tumor progression through miRNA interactions, which makes them attractive candidates for non-invasive diagnostic tools and targeted medical approaches. Through their miRNA sponge activity, they regulate gene expression together with their ability to modulate RNA-binding proteins, affect mRNA stability and impact the PTEN/PI3K/Akt and MAPK pathways that enable tumor growth and survival. The study demonstrates how circRNAs function through multiple mechanisms in endometrial cancer development, yet indicates their value as therapeutic targets for disease management. The paper explores how recent developments utilize circRNA strategies to combat chemoresistance alongside improving targeted therapy research. Future research must focus on optimizing detection methods while developing standardized diagnostic procedures and performing large-scale validation studies to establish clinical utility.

## Introduction

Endometrial cancer is among the most common malignant tumors of the female genital tract. Its epidemiological characteristics vary among different regions and populations. Endometrial cancer has, however, risen in the last few years and is prevalent in the current society, more so in developed countries. New cases of endometrial cancer remain estimated at 60,000 every year, therefore making it the most common malignant tumor of the female genital tract in the United States [1]. Owing to the increase in the economy and changes in lifestyle in China, the incidence of endometrial cancer has gradually risen and has become one of the most serious diseases that endangers women’s health [2]. Epidemiologically, endometrial cancer is predominant in post-menopausal women, particularly those within the age bracket of 50–69 years. Nevertheless, it has been observed in recent years that the proportion of young women with endometrial cancer is also on the rise. This may be associated with observed changes in life, rising obesity, and the use of hormone replacement therapy (HRT) [3, 4]. At the same time, it is stated that metabolic diseases, including hypertension, diabetes, and polycystic ovary syndrome (PCOS), are also risk factors for endometrial cancer [5]. These diseases often come with hormonal dysfunctions and metabolic derangement, which, in a way, would culminate in endometrial cancer [6]. Besides, the presence of certain family genetic factors was also found to have a certain level of influence on the development of endometrial cancer. It is reported that 5–10% of women who develop endometrial cancer have a family history of this disease; however, women with endometrial cancer is generally rare among patients with hereditary nonpolyposis colorectal cancer syndrome and Lynch syndrome [7].

Endometrial cancer is understood as a multifactorial disease, and the details of its pathogenesis have not been explained yet [8]. However, continuous in-depth study of molecular biology and genetics has given people a clearer understanding of pathogenesis [9]. Thus, multiple genes and abnormal molecular expression are primarily associated with the occurrence of endometrial cancer. Of them, the most important include the changes in gene PTEN, PI3K-AKT-mTOR pathway, and p53 [10, 11]. The factors affecting the pathophysiology of this cancer include estrogen, in addition to the physiological and mechanical changes. Endometrial hyperplasia arises from excessive endometrial tissue growth due to prolonged exposure to estrogen compounds and may predispose one to endometrial cancer [12, 13]. In any normal condition, estrogen and progesterone hormones work in harmony to stimulate the growth and differentiation of the endometrial cells. However, it weakens when the action of estrogen is relatively enhanced, and the effect of antagonization by progesterone is the opposite; the estrogen stimulates the proliferation and cancer transformation of the endometrium [14, 15]. Endometrial cancer is also known to be associated with the uncontrolled activation of specific cell signaling systems. For instance, the Wnt/β-catenin signaling system acts continuously within such activities as migration, differentiation as well as cell proliferation. This signaling pathway, when dysregulated, will cause *β*-catenin protein to be present in the cytoplasm of the cell and activate downstream target genes involved in cell proliferation and cancer transformation [16].

It is suggested that endometrial cancer early detection and treatment would enhance the survival rate and quality of life of the patients. Patients with stage I endometrial cancer naturally undergo surgical treatment and then receive comprehensive treatment measures, radiotherapy, chemotherapy, or HRT, depending on the individual situation, and the survival rate after 5 years is more than 90% [17]. Nonetheless, it becomes poor when the endometrial cancer patient has reached the advanced stage because cancer cells spread and metastasize, making the treatment not so effective and thus the low 5-year survival.

Endometrial cancer molecular mechanism associated with Circular RNAs (circRNAs) has been addressed, as circRNAs are presupposed to contain diagnostic and therapeutic values in the development and chemoresistance of endometrial cancer. These covalently closed RNA molecules appear to be stable biological entities with strong regulatory capacities that play diverse roles in cancer related events. CircRNAs can interact with targets in endometrial cancer and change the expression of related cellular signaling pathways, and these have primarily acted as microRNA (miRNA) sponges. A clear example in this regard is circ_0007534, which promotes chemoresistance and cancer progression through the miR-625-ZEB2 axis. Consequently, this mechanism is associated with chemo-resistance, whereby circRNAs help cancer cells escape from chemotherapy induced-apoptosis [18]. Furthermore, the circRNAs in exosomes, including circulating extracellular vesicles, can be delivered between cells to mediate chemoresistance as well as remodel the tumor microenvironment. These results underscore their position not just in relation to their part in cancer biology but also while defining targets around resistance pathways.

CircRNAs can also be used as diagnostic markers for endometrial cancer. Because they do not alter rapidly in blood and other biofluids, they can be ideal for establishing non-invasive diagnostic and prognostic biomarkers. It has been found that circRNAs can effectively distinguish the cancerous state from the normal state and classify chemosensitivity [19]. From the therapeutic point of view, circRNA-miRNA interference provides novel approaches to molecular targeting in precision medicine from a new angle. For example, CDR1as, which has been proven to help reduce chemoresistance, can inhibit miR-1299 and improve the efficacy of cisplatin-containing chemotherapy in gynecological cancers [20]. These findings make it possible to design and develop medications and treatments for diseases based on circRNA that can be used in association with the existing treatments.

## CircRNA basic theory

### Definition and discovery

circRNAs are a subgroup of endogenous noncoding RNAs (ncRNAs). However, the circRNAs, which are the main focus of this article, have unique and distinct circular RNA characteristics and do not contain a 5′ terminal cap structure or a 3′ polyadenosine tail, but they are linked covalently in a circular pattern [21]. At that time, scientists studying viruses and plant RNAs accidentally discovered some RNA molecules with circular structures [21]. However, due to technological limitations at the time, research on these circRNAs progressed slowly, as they were considered abnormal products in RNA splicing processes and did not receive sufficient attention [22]. A growing number of circRNAs have been identified as a result of the ongoing development of molecular biology tools, particularly the rise of high-throughput sequencing technology [23]. A number of researchers have proved that circRNAs are highly expressed in different species of animals, plants, and microorganisms. For instance, as stated before, there are 10,000-plus newly discovered circRNAs in human cells. These circRNAs are unique in size and nucleotide sequence, and they can be derived from different locations in a gene: exonic, intronic, or from the intervening regions.

### Biological function

**Fig. 1:**
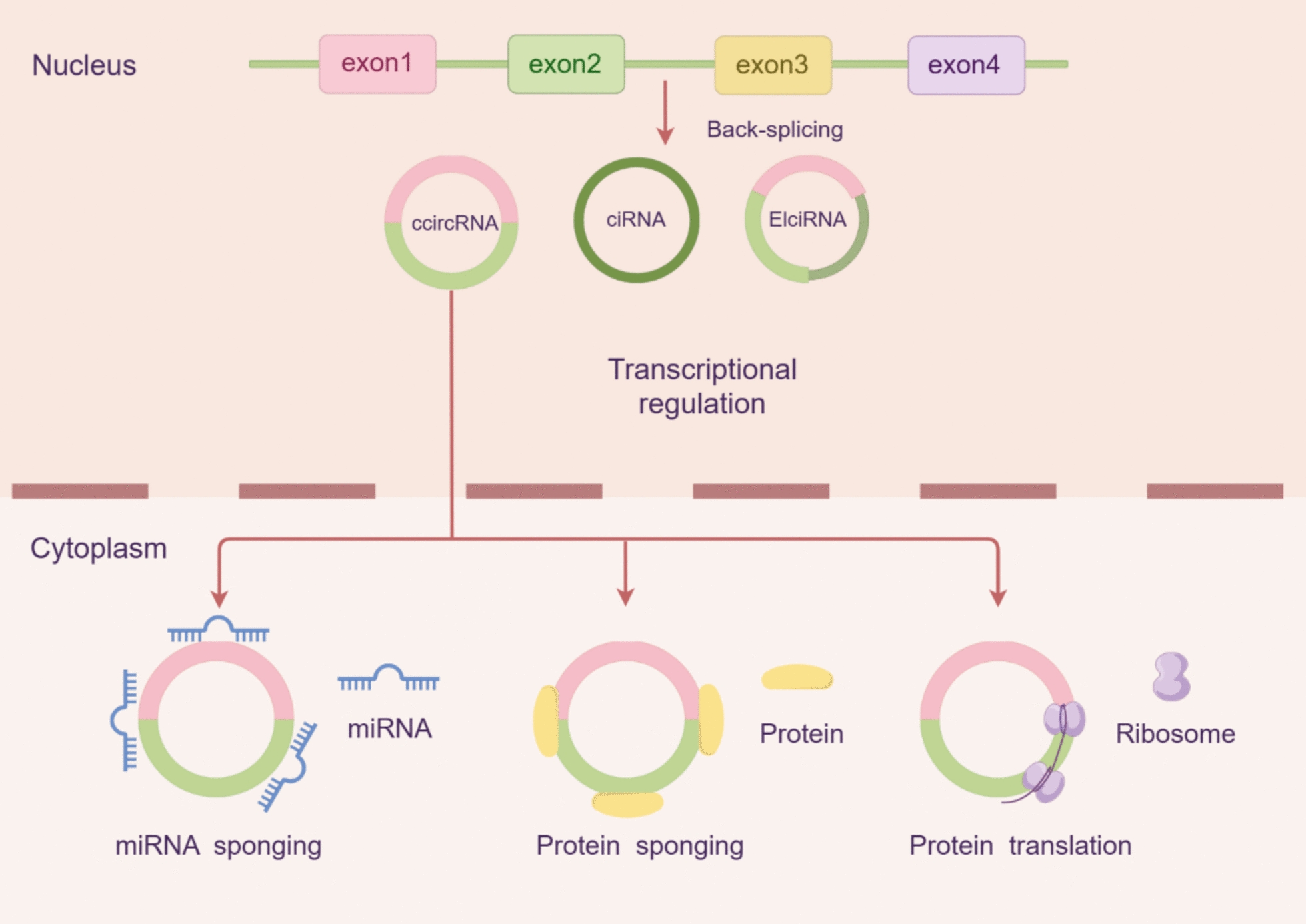
Effect of circRNA on protein and miRNA. This figure depicts the biogenesis of circRNAs via back-splicing and their functional roles in the nucleus and cytoplasm. In the cytoplasm, circRNAs regulate gene expression by sponging miRNAs and proteins or undergoing translation into peptides

These circRNAs have diverse biological functions and are vital for metabolic processes, cell proliferation, development, and disease initiation or development [24]. CircRNAs have the ability to sequester miRNAs. Over 70% of circRNAs contain multiple microRNA response elements (MREs) to which miRNAs can directly bind and thus neutralize the miRNA-mediated regulation of their target genes [25]. For example, the first circRNA with miRNA sponge activity, CDR1as, interacts with miR-7 at more than 70 sites to control the expression of genes by miR-7. Hence, some members of the circRNA family, including circ-ITCH (circular RNA itchy E3 ubiquitin protein ligase) and circ-HIPK3, have also been described to possess miRNA sponge activities. This current miRNA acts like a sponge function, which can decrease gene expression quantities, modulate the intracellular activity of miRNA, and participate in a large scale of physiological and pathological events occurring intracellularly (Fig. 1).

Since circRNAs are directly associated with RNA binding proteins (RBPs), the capacity to serve as a protein sponge might be a reality. For instance, Circ-Foxo3 can heterodimerize with CDK2 and p21 and can interact with a number of proteins, inhibiting cell cycle and G1–S phase progression [26]. The activity of RNA–protein complexes can be affected by some circRNAs' ability to bind to proteins and form circRNA-protein complexes (circRNPs). CircRNAs can also control the stability of mRNA. CircRNAs have the ability to control gene expression by binding to mRNA and altering the pace at which mRNA degrades. Ci-ankrd52 and Ci-sirt7 function as cis-acting elements to control the transcriptional activity of RNA polymerase II, demonstrating that circRNAs may also control gene transcription. Although most circRNAs are considered endogenous noncoding RNAs, a few circRNAs have been found to be involved in protein translation, such as the core circRNA of hepatitis D virus (HDV) and the HDV antigen, which can be jointly translated into the same protein.

### Association with cancer

The data in the last several years have shown that circRNAs play crucial roles in carcinogenesis, invasion, metastasis, and the incidence of cancer [24]. CircRNAs are endogenous non-coding RNAs known for having closed loop structures, formed by something called “back-splicing,” a non-standard splicing process. Being devoid of cap and tail structures at the ends, circRNAs can last much longer than other RNA types and are more protected from enzymes that break them. At first, scientists thought these structures were just mistakes in the DNA, but today we know that they serve functions in cells and gene regulation [25]. miRNA sponging is a significant way that circRNAs function in the body.

One tumor-suppressing gene, miR-7, for instance, can suppress the production of carcinogenic proteins in tumor-related signaling pathways, preventing tumor formation and recurrence through a variety of mechanisms [27]. CDR1as, acting as a sponge for miR-7, competitively absorbs miR-7 and suppresses its expression, thereby affecting the biological processes of tumor occurrence, development, invasion, and metastasis by regulating the expression of target genes. For example, ciRS-7 (also known as CDR1as) works as a competitive endogenous RNA (ceRNA) and interacts with more than 70 molecules of miR-7. It can efficiently hold on to miR-7 and manage the effects of downstream cancer targets, as mentioned by Memczak et al. [25]. This sponging capacity allows circRNAs to modulate the expression of multiple genes, thereby influencing diverse cellular processes such as proliferation, apoptosis, and metastasis.

Similarly, circ-ITCH is significantly downregulated in tumor tissues [28]. It can regulate the activity of relevant miRNAs, upregulate ITCH levels, further affect the stability of proteins through single ubiquitination pathways or polyubiquitination protein degradation pathways, and participate in the regulation of p63 gene expression, thereby promoting cell terminal differentiation and exhibiting anti-cancer effects. CircRNAs regulate the expression of tumor proto-oncogenes.

Despite being involved in the nucleus of the cell to act as a sponge for the miRNA, ciRNAs have the ability to regulate proto-oncogene expression patterns. For instance, ci-ankrd52, overexpressed in the cell nucleus and significantly dispersed, can upregulate the proto-oncogene [29]. When certain circRNAs are knocked out, the expression of their proto-oncogenes decreases, and intronic circRNAs can also positively regulate the transcription of RNA polymerase II. circRNAs can also serve as tumor markers. For example, the plasma and tissues of individuals with gastric cancer exhibit a marked downregulation of hsa_cir_002059 [30]. Gastric cancer, for example, overexpresses CircPVT1, and this increases cell growth by removing miR-125 from the cells’ environment [31]. circRNAs expression level varies according to clinical pathological features such as TNM staging and the presence of distant metastasis [32]. In addition, hsa_circ_0001649 is significantly downregulated in hepatocellular carcinoma tissues. The expression level of hsa_circ_0001649 is considerably lower in larger tumor tissue or in the presence of a cancer thrombus [33]. This indicates that circRNAs serve as new tumor biomarkers that could help in the diagnosis and prognosis of different forms of cancer.

Because they are consistent in the blood and in cancer tissue, can be found in exosomes, and show correct patterns for detection, they may be useful in early cancer detection procedures. For example, improper activation of circSMARCA5 could promote glioblastoma growth and might be used as a way to classify patients by prognosis [34]. Besides, new research indicates that circRNAs, in special cases, can be translated into useful peptides. Scientists have discovered that internal ribosome entry sites (IRES) and open reading frames (ORFs) in CirRNAs make them capable of translation, which goes against the traditional belief that they do not code for proteins. For instance, evidence from Legnini et al. suggests that Circ-ZNF609 serves a wide regulatory function by being involved in myogenesis [35].

## Expression of circRNA in endometrial cancer

### Differences in expression patterns

CircRNA expression in endometrial cancer is substantially different from that in healthy uterine tissue [24]. It has been demonstrated that circRNA expression levels exhibit complex changes in endometrial cancer tissues and cells. Some circRNAs are upregulated in endometrial cancer tissue. For example, Chen et al. compared tumor tissues from endometrial cancer patients with normal endometrial tissues and identified 22 circRNAs that were upregulated in endometrial cancer tissue [36]. These enhanced circRNAs might be involved in the promotion of endometrial cancer in multiple ways. CircRNAs can, for instance, function as a miRNA sponge to affect cellular biological events, namely invasion, proliferation, and metastasis in cancer cells [25]. Conversely, several circRNAs are downregulated in endometrial cancer tissue. In the same study by Chen et al., it was found that 98 circRNAs were downregulated in endometrial cancer tissue [36]. Endometrial cancer tumor tissue lacked the circRNAs RP11-255H23.4 and HSPG2, which are only expressed in healthy endometrial tissue. This downregulation may weaken their regulatory effects on relevant biological processes, making it difficult to effectively inhibit the growth and development of cancer cells. For example, HSPG2 is mainly involved in encoding heparan sulfate proteoglycans, which influence the growth or regeneration of endothelium by binding to growth factors on the basement membrane. Loss of its expression may affect the inhibitory effect on the progression of endometrial cancer [37]. Findings also demonstrate the association between endometrial cancer clinical/pathological characteristics and circRNA expression patterns. For instance, in their study of endometrial cancer patients’ serum, Xu et al. discovered that the patient’s serum contained more extracellular vesicles than healthy persons, 209 circRNAs were upregulated, and only 66 circRNAs were downregulated [38]. Furthermore, endometrial cancer patients showed significantly raised levels of hsa_circ_0109046 and hsa_circ_0002577, and their serum levels were at least twofold [39]. This indicates that circRNA expression may be closely related to clinical and pathological features of endometrial cancer, such as FIGO stage and metastatic traits, and therefore provides novel information for the aetiological, diagnostic and therapeutic approaches to endometrial cancer [24]. Table 1 shows the important differences in circRNAs expression levels between endometrial cancer tissues and normal endometrial tissues.

**Table 1:** Significant distinctions in the profiles of tested genes involved in endometrial cancer and normal endometrial tissues

Study	Specific circRNAs	Possible mechanisms	Possible effects	Expression status (upregulated/downregulated)	References
Yang et al. [40]	circRNA-0115118	Regulates the miR-138–1-3p/WDFY2 axis	Influences endometrial function	Upregulated	[40]
Jiao et al. [41]	Hypoxia-related circRNAs	Links hypoxia signatures to survival outcomes in EC	Prognostic biomarker	NR3C1: Upregulated IL-6: Downregulated SRPX: Downregulated	[41]
Li et al. [42]	hsa_circRNA_079422, miR-136-5p	circRNA-miRNA coexpression in cancer	Potential role in tumor growth and spread	hsa_circRNA_0794: Downregulated miR-136-5p: Upregulated	[42]
Dakal et al. [43]	hsa-miR-433-3p, hsa-miR-188-3p	Analysis of competing endogenous RNA interactome	Tumor suppressor and oncogenic pathways identified	hsa-miR-433-3p: Upregulated hsa-miR-188-3p: Upregulated	[43]
Yin et al. [44]	circFN1, circGLIS2	Interaction of circRNA, lncRNA, and mRNA in endometriosis	Potential pathway insights for cancer	circFN1: Upregulated circGLIS2: Upregulated	[44]
Liu et al. [45]	circTNFRSF21	Regulates miR-1227-MAPK13/ATF2 axis	Promotes endometrial carcinoma pathogenesis	circTNFRSF21: Upregulated	[45]
Ye et al. [46]	hsa_circ_0039569, hsa_circ_0001610	Grade 3 EC circRNA profiles differ significantly	Predictive of cancer stage	hsa_circ_0039569: Upregulated hsa_circ_0001610: Upregulated hsa-miR-542-3p: Downregulated let-7c-5p: Downregulated	[46]
Xu et al. [38]	Serum circRNAs	Role in extracellular vesicle-mediated gene expression	Diagnostic potential	hsa_circ_0109046: Upregulated hsa_circ_0002577: Upregulated	[38]

### Identification of key circRNA

**Fig. 2:**
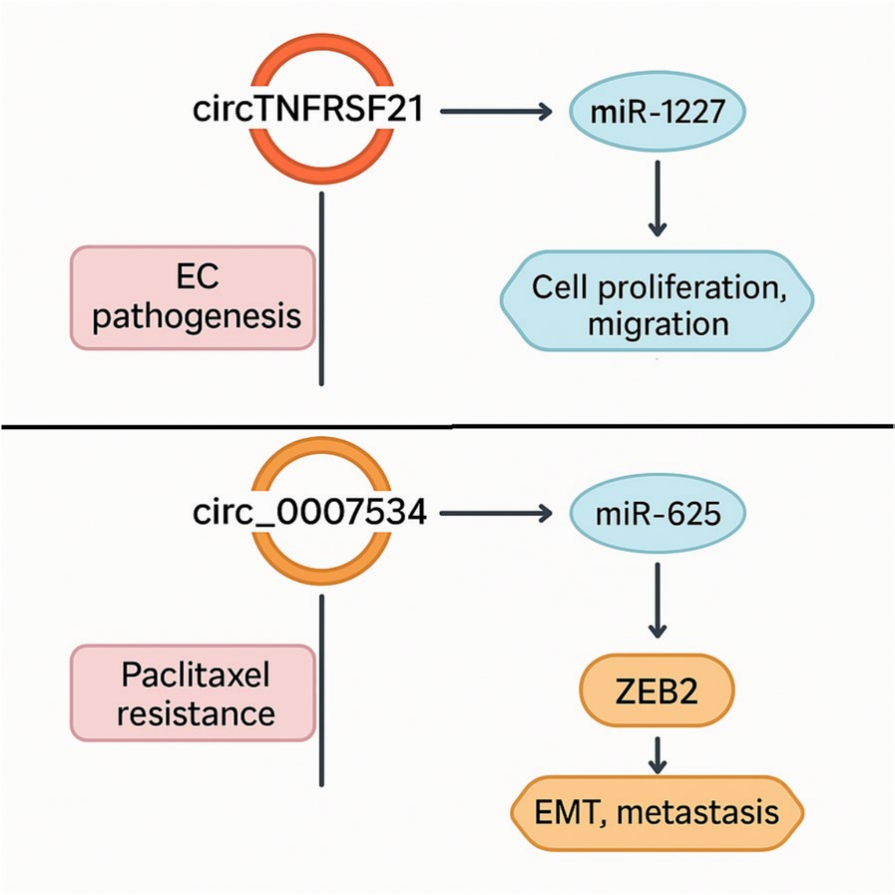
Key circRNAs and their regulatory axes in endometrial cancer. circTNFRSF21 promotes EC pathogenesis by sponging miR-1227, enhancing cell proliferation and migration. circ_0007534 contributes to paclitaxel resistance by targeting miR-625, leading to ZEB2 upregulation and activation of EMT and metastasis pathways

Thorough knowledge of the disease process and the creation of novel diagnostic and treatment approaches depend heavily on the identification of important circRNAs among the numerous circRNAs that are intimately linked to the onset and progression of endometrial cancer [24]. circ-ITCH is a widely studied key circRNA, which contains the exon of ITCH and plays a crucial role in various types of tumors [47, 48]. Because circ-ITCH competes with miRNA-17 and miRNA-224 in endometrial cancer, its target genes, p21 and PTEN, express themselves differently. Once the PTEN gene mutates or loses activity, the phosphatase becomes inactive, leading to a loss of inhibition of cell proliferation, causing cells to undergo malignant transformation and ultimately leading to the formation of endometrial cancer [49, 50]. Although this axis is well-characterized, other circRNAs, such as circTNFRSF21 and circZNF91, also modulate oncogenic pathways in endometrial cancer. circ-ZNF91 is also a key circRNA. In endometrial cancer tissues, circ-ZNF91 shows a negative correlation with the expression of miRNA-23B and miRNA-122A2, suggesting that this circRNA may act as a miRNA sponge to inhibit the expression of miRNA-23B and miRNA-122A2 in cells [51]. Current domestic and foreign literature has confirmed the association of the above two miRNAs with the occurrence of various human tumors, and due to the different types of tumors, their target genes vary. They play roles in either inhibiting or inducing cancer during the occurrence and development of different tumor tissues [52, 53]. Estrogen receptor 1 (ESR1) plays a key role in estrogen-dependent endometrial cancer. Studies have found that 11 ESR1 circular isoforms are expressed in endometrial cancer tissues, and detecting tumor-specific circular isoforms of the ESR1 gene may become a molecular marker for early diagnosis of endometrial cancer [54]. Figure 2 illustrates some of the important interactions between circRNAs, miRNAs, and relevant mRNAs linked to the development and progression of endometrial cancer.

### Functional verification methods

To verify the function of circRNA in endometrial cancer, a variety of experimental methods need to be employed (Table 2). At the cellular level, commonly used approaches include cell transfection and gene silencing techniques. Then, the vectors of circRNA overexpression or RNAi are transfected into endometrial cancer cells to observe the alteration in biological functions, including cell growth, apoptotic cell death, migration, and invasion [24]. For instance, by using stably transfected endometrial cancer cells, the integration of overexpression vectors such as circ_0007534 can demonstrate their respective roles in cancer suppression or chemoresistance. A nude mouse model is employed where endometrial cancer cells that are transfected with circRNA overexpression vectors or interfering RNA are used to determine the effects of the vectors or interference on the tumor growth, including alteration in tumor volumes or weight [55]. For instance, nude mice are used to examine the role of circ-ZNF91 in vivo; endometrial cancer cells with interfering RNA for circ-ZNF91 are used to be transplanted into nude mice and the development of tumors. Besides cell and animal experiments, the function of circRNA can also be confirmed by molecular biology approaches. For example, the content of circRNA, the corresponding miRNA, and mRNA are identified using quantitative reverse transcription polymerase chain reaction (qRT-PCR) to detect the RNA level, and the protein level is determined using Western blot to explain the regulatory role of circRNA in gene expression [56]. For example, in the study of the regulatory role of circ-ITCH on the PTEN gene, qRT-PCR can measure the changes in the expression level of PTEN at the mRNA level, while the Western blot assay can be used for measuring the changes in PTEN at the protein level. In addition, bioinformatics methods can be used to predict target genes and the function of circRNA. The potential target gene can be predicted by studying the sequence features of circRNA and miRNA binding sites, which offers theoretical guidance for the following experimental test. For instance, the targets and potential pathways of circ-ZNF91 can be computationally predicted by using the bioinformatics method, and further, it is possible to prove or disprove these predictions through a series of experiments.

**Table 2:** Functional verification methods

Experimental Level	Experimental Method	Specific Operation
Cellular Level	Cell Transfection	Construct overexpression vectors or inhibitory RNAs (RNAi) for circRNAs and transfect them into endometrial cancer cells
	Gene Silencing Technique	Use specific methods to reduce or silence the expression of circRNAs
Animal Level	Nude Mouse Tumorigenesis Experiment	Inoculate endometrial cancer cells transfected with circRNA overexpression vectors or inhibitory RNAs into nude mice and observe tumor growth
Molecular Biology Level	qRT-PCR	Detect the expression levels of circRNAs, related miRNAs, and mRNAs
	Western Blot	Detect the expression levels of related proteins
Bioinformatics Level	Bioinformatics Analysis	Analyze the sequence characteristics of circRNAs and the binding sites of miRNAs to predict target genes and signaling pathways

Besides experimental approaches, analysis of bioinformatics is also crucial in the study of circRNA. A number of web databases and software programs are used to predict circRNA structure, expression, and function. As an example, CircInteractome [57] and CircBank [58] allow exploring circRNA-miRNA and circRNA-RBP interactions, respectively, to understand regulatory networks. Specifically, tools such as CIRI2 ([59] can help to perform precise circRNA identification after RNA-seq data, whereas miRanda [60] and TargetScan [61] are widely used to find possible miRNA binding sites on circRNAs. Such resources facilitate the structure and validation of functionally relevant circRNAs and thus aid discovery of novel therapeutic and diagnostic targets in endometrial cancer.

## CircRNA influences the mechanism of endometrial cancer progression

### Cell proliferation regulation

In endometrial cancer, circRNA is essential for controlling cell division. That is, cell proliferation is one of the major events in the formation and progression of tumors, and circRNA can mediate the impacts on endometrial cancer cell proliferation in different pathways [24]. The experiment results revealed that CircRNA could work as a miRNA sponge to regulate the already identified genes related to cell proliferation. For example, circZNF91 and circTNFRSF21 contribute to dysregulation of proliferation pathways through interaction with tumor-suppressive miRNAs. Studies have shown that PTEN, as a tumor suppressor gene with phosphatase activity, can inhibit tumor development from multiple aspects, such as promoting tumor cell apoptosis, inhibiting cell cycle progression, suppressing tumor cell proliferation, invasion, and metastasis, inhibiting tumor tissue angiogenesis, and stabilizing the immune system [62]. In addition, circRNA can influence cell proliferation through interactions with RBPs. For instance, the recently identified circ-Foxo3 can directly bind with several proteins that associate with CDK2 and p21 to suppress the cell cycle and block the G1 to S phase progression [63]. In endometrial cancer cells, such interaction may be altered, leading to excessive cycling of the cell division cycle and encouraging cell division. Some circRNAs can also influence cell proliferation by regulating cell signaling pathways. The PI3K/Akt, MAPK, and other signaling pathways, for example, are crucial for cell division, survival, and proliferation and may be activated or inhibited by certain circRNAs. When these signaling pathways are aberrantly activated or inhibited, it may lead to uncontrolled proliferation of endometrial cancer cells.

### Impact of apoptosis

Apoptosis is a type of programmed cell death that is most vital for controlling cell populations and ensuring proper tissue growth [64]. Alteration in apoptotic regulation is among the critical processes that lead to endometrial cancer development, and this type of circRNA can affect apoptosis through different pathways. Some circRNAs can control cell apoptosis by modulating apoptotic-related gene expression. Reduction in circ-ITCH expression raises miRNA-17 and miRNA-224 activity, which in turn lowers p21 and PTEN expression and prevents tumor cells from dying. Some circRNAs can also affect apoptosis by modulating the apoptosis signaling pathways. For instance, certain circRNAs can activate or inhibit the activity of the Caspase family proteins, which are key proteases in the process of apoptosis, and their activation can trigger apoptosis [65]. In addition, circRNAs can influence apoptosis by interacting with apoptosis-related proteins. For example, circ-Foxo3 interacts with the p53 protein, enhancing the stability and activity of the p53 protein, thereby promoting apoptosis [26]. There is evidence that in endometrial cancer cells, circ-Foxo3 may be deregulated, and this might negatively affect the activity of the p53 protein, which inhibits apoptosis.

### Epithelial–mesenchymal transition

Epithelial–mesenchymal transition (EMT) is a process that results in the change of epithelial cells from a strictly polarized tissue structure connected to neighbor cells to mesenchymal cells. Based on the abovementioned CircRNAs' effects on the EMT process and their involvement in several pathways, EMT is crucial to tumor invasion and metastasis in endometrial cancer [56]. CircRNAs can thus regulate the EMT process by modulating the expression of genes associated with EMT. For example, several circRNAs have the capacity to modulate the expression of genes related to EMT, such as *N*-cadherin, Vimentin, and *E*-cadherin [66]. In the case of *E*-cadherin siRNA-transduced cells, down-regulation of *E*-cadherin deprives epithelial cells of their intercellular connections and causes EMT. There are two markers that indicate mesenchymal cells; raising their expression levels leads to mesenchymalization of the cells. It has been possible to establish direct correlations between the expression changes of specific circRNAs and EMT associated genes in endometrial cancer, implying that circRNAs could interact with the EMT process by managing the expression of genes related to EMT [18, 67]. CircRNAs can also participate in the regulation of the EMT process as regulators of signaling pathways. For instance, specific circRNAs are known to act as activators or repressors of signaling cascades involved in the EMT, for instance, Wnt/β-catenin and TGF-*β*. Abnormal activation or inhibition of these signaling pathways may induce EMT in endometrial cancer cells, further promoting tumor invasion and metastasis.

### Angiogenic activity

Angiogenesis is one of the key processes in tumor growth and metastasis, where tumor cells rely on newly formed blood vessels to obtain nutrients and oxygen while disposing of metabolic waste. In addition, circRNAs are essential for the angiogenesis of endometrial cancer [68]. CircRNAs can influence angiogenesis by regulating the expression of genes related to blood vessel formation. For example, circ-0000437, which contains a functional peptide called CORO1C-47aa. This peptide was demonstrated to inhibit angiogenesis in endometrial tumors through the inhibition of VEGF-A secretion, a key pro-angiogenic factor. It shows a peptide-coding function of a circRNA to restrain blood vessel development in cancer tissue [69]. circWEE1 (circ_003390) is another highly studied circRNA that was found to spur angiogenesis in EC by boosting VEGF/VEGFR signaling, which is a canonical pathway in blood vessel formation. This axis is controlled by CircWEE1, which interacts with eukaryotic translation initiation factor 4A3 (EIF4A3) to stimulate cell proliferation and migration [70].

In addition, circRNAs can also influence angiogenesis by regulating signaling pathways related to blood vessel formation. For instance, some of the circRNAs can act as activators or repressors of signaling pathways, which include VEGF and angiopoietin signaling pathways that are involved in angiogenesis [71, 72]. Abnormal activation or inhibition of these signaling pathways may lead to abnormal blood vessel formation in endometrial cancer tissues, thereby affecting the growth and metastasis of tumors.

**Fig. 3:**
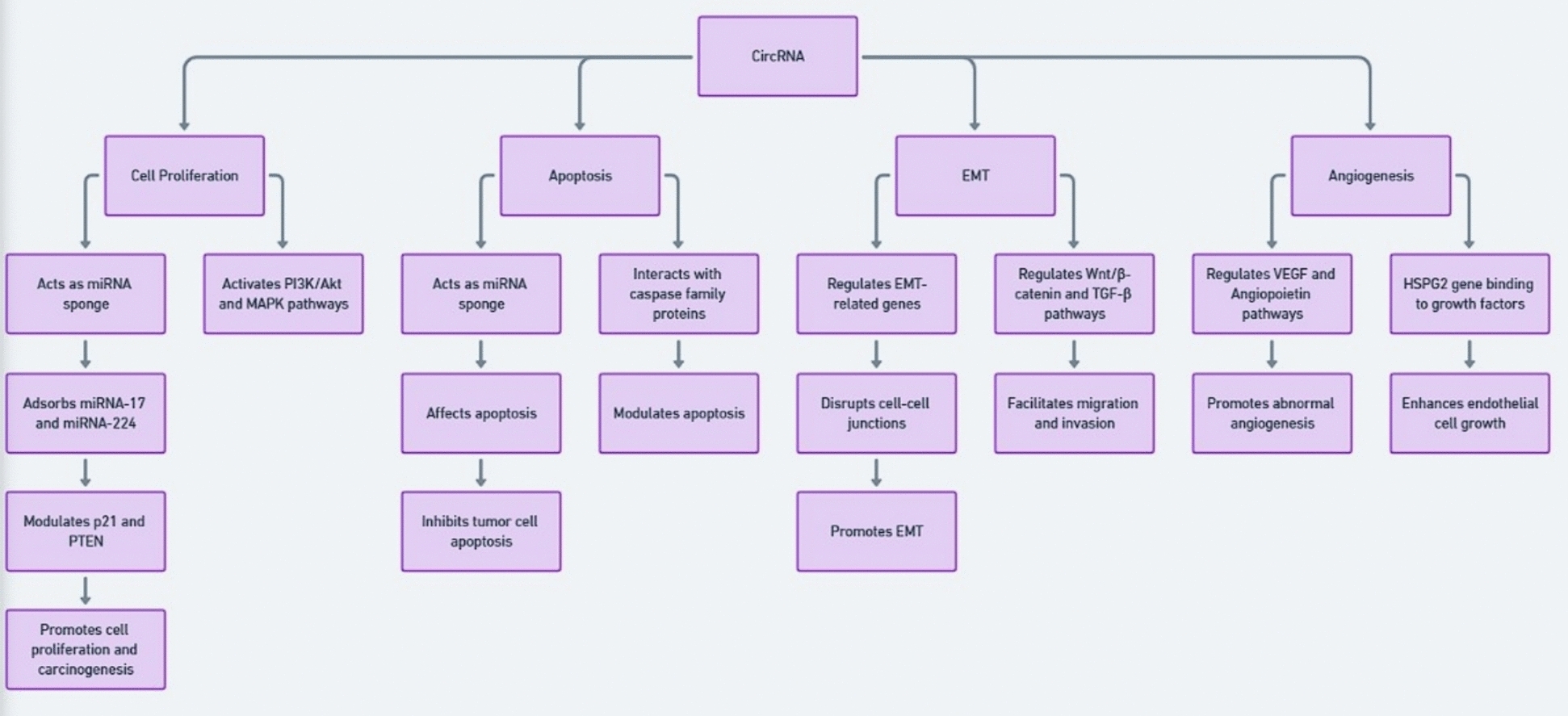
CircRNA influences the mechanism of endometrial cancer progression. CircRNAs promote cell proliferation by sponging miRNAs and activating PI3K/Akt and MAPK pathways. They inhibit apoptosis by targeting caspase proteins and miRNAs. CircRNAs also regulate epithelial–mesenchymal transition (EMT) by modulating EMT-related genes and Wnt/β-catenin or TGF-β signaling, thereby enhancing invasion. In addition, circRNAs drive angiogenesis through VEGF/angiopoietin signaling and HSPG2-mediated endothelial growth

The schematic in Fig. 3 demonstrates how circRNAs manipulate various endometrial cancer molecular pathways through gene control and miRNA sponge effects, and protein regulatory mechanisms leading to tumor growth and metastasis occurrences.

## Study on the relationship between circRNA and chemoresistance in endometrial cancer

### Mechanisms of drug resistance

**Fig. 4:**
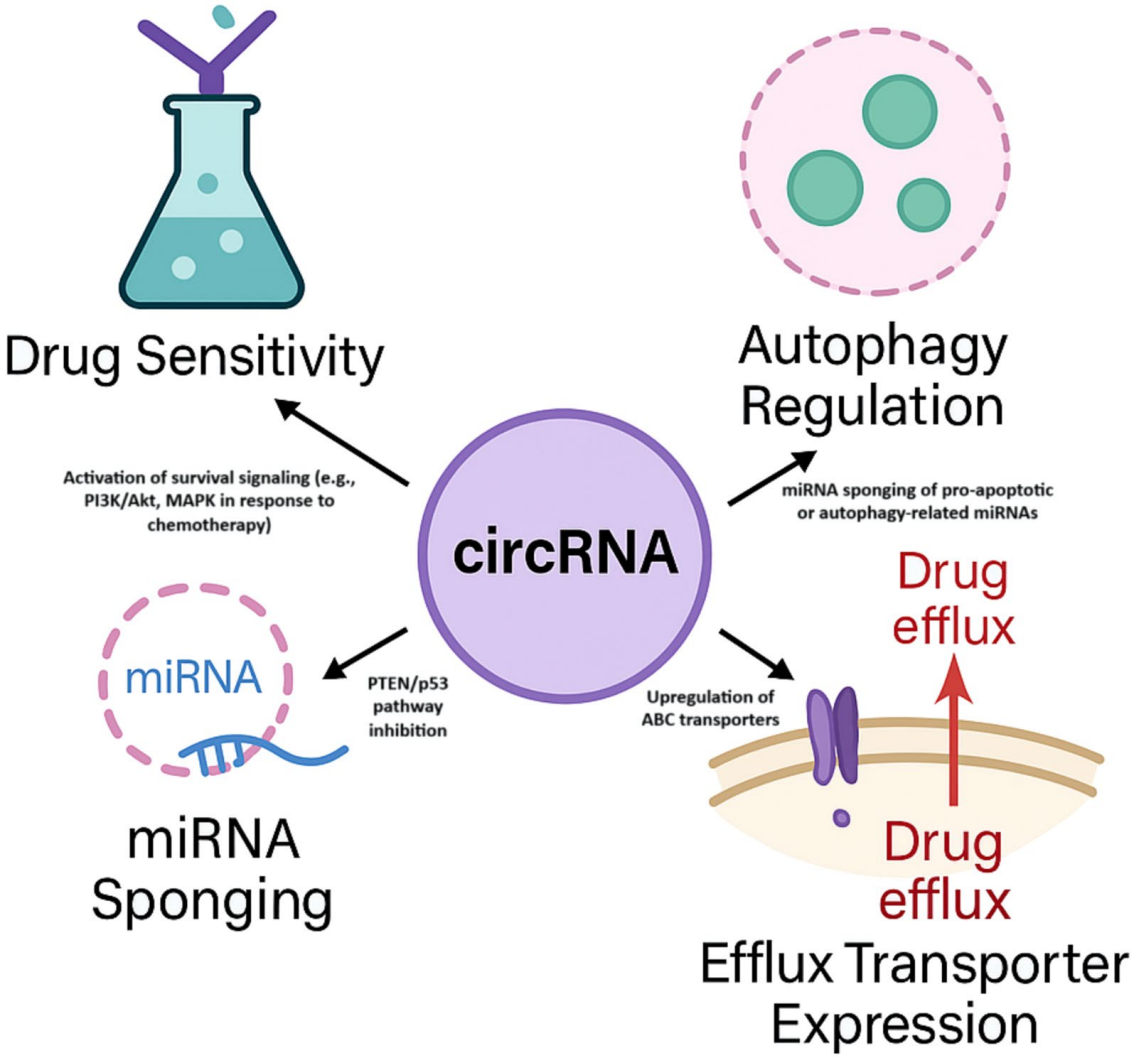
circRNA-mediated chemoresistance in endometrial cancer

One of the main obstacles to endometrial cancer’s clinical treatment is its resistance to chemotherapy. In this process, circRNA is involved in mediating drug resistance, and the anti-drug resistance mechanisms are manifold and intricate, and circRNA can act as a miRNA sponge. For instance, certain circRNAs can through the process of sponging the stated miRNA, meaning that it will control the impact of the miRNA on its target genes [24]. In endometrial cancer, certain miRNAs are closely associated with the sensitivity to chemotherapy drugs [73]. When circRNAs are dysregulated, they influence the functions of miRNAs; consequently, the levels of related target genes change, and drug resistance occur in cancer cells. For example, studies revealed that circ_0007534 is known to promote paclitaxel resistance by sponging miR-625 and upregulating ZEB2, contributing to EMT and chemoresistance. When inactivated or expressed improperly, PTEN, a tumor suppressor gene with phosphatase activity, is directly linked to the growth and medication resistance of tumor cells. Mutations or inactivation of the PTEN gene can lead to phosphatase inactivation, thereby losing the ability to inhibit cell proliferation, causing cells to become malignant and inducing the occurrence of endometrial cancer, while also potentially decreasing the sensitivity of cancer cells to chemotherapy drugs [23]. circRNA can also influence drug resistance by regulating signaling pathways within cells. Some circRNAs can regulate signaling pathways related to cell survival, apoptosis, and drug metabolism, allowing cancer cells to adapt to the pressure of chemotherapy drugs and develop resistance. The PI3K/Akt signaling system, for instance, may be activated by certain circRNAs, improving cancer cells' capacity to endure and multiply in the presence of chemotherapy medications [24]. In addition, circRNA can impact the expression and activity of drug-metabolizing enzymes, altering the metabolism and distribution of chemotherapy drugs within cells, thereby reducing the efficacy of the drugs [74]. The engagements of circRNA with the tumor microenvironment (TME) are also involved in controlling the drug resistance mechanisms. Noncancerous cells within the tumor, including tumor-associated fibroblasts and immune cells, as well as components of the extracellular matrix within the TME, can affect the behavior of the cancer cells and their response to chemotherapeutic agents [75]. circRNA can modulate cell–cell signal and/or cell–cell signaling pathway, altering the structure and function of TME and the drug resistance of cancer cells. That is, circRNAs may directly or indirectly modulate secretion and signaling of cytokines, recruit immunosuppressive cells into the tumor microenvironment, suppress immune system’s cytotoxic activity towards cancer cells, and support drug resistance of cancer cells. These resistance strategies are summarized in Fig. 4, which visually depicts the primary circRNA-mediated mechanisms contributing to chemoresistance in endometrial cancer.

### circRNA as biomarkers

Given its stability and specific expression patterns in cells, circRNA has the potential to become a novel marker for chemoresistance in endometrial cancer [24]. Several investigations have revealed the factors that define the degrees of circRNAs in endometrial cancer tissues and how blood and chemoresistance relate to each other. Certain circRNAs were discovered to express themselves much differently in resistant tumor tissues than in sensitive tumor tissues when endometrial cancer tumor tissues and surrounding tissues were evaluated at the tissue level [76]. For example, Chen et al. identified 11 circular isoforms of ESR1 in endometrial cancer tissues, detecting tumor-specific circular isoforms of the ESR1 gene as a molecular marker for early diagnosis of endometrial cancer [36]. Similarly, in exploring chemoresistance, certain specific circRNAs may show specific high or low expression in resistant tumor tissues, providing a basis for assessing tumor resistance by measuring the expression levels of these circRNAs. The function of CircRNA as a miRNA sponge also supports its role as a biomarker. Numerous circRNAs can competitively absorb miRNAs, thereby influencing the regulation of target genes by miRNAs and participating in the biological regulation of endometrial cancer. Circ-ITCH, for instance, has the ability to competitively absorb miRNA-17 and miRNA-224, which results in differential expression of their target genes, p21, and PTEN, and influences the development and incidence of endometrial cancer [77]. circRNA as a marker also has certain advantages. Compared with traditional tumor markers, circRNA has higher stability and specificity. Owing to its circular structure, circRNA is not easily degraded by exonucleases, allowing it to remain stable in biological samples such as blood and saliva, facilitating detection [30]. A more realistic representation of the biological features of tumors is provided by the strong relationship between the expression pattern of circRNA and the formation, progression, and chemoresistance of tumors.

### Overcoming drug resistance strategies

The association between circRNA and chemotherapy resistance in endometrial cancer has significant clinical implications. One effective strategy is targeted therapy based on circRNA. Since circRNA plays an important role in drug resistance mechanisms, interfering with the function or expression of circRNA by designing specific targeted drugs or nucleic acid molecules can reverse the drug resistance of cancer cells [24]. For example, using RNAi or antisense oligonucleotides (ASO) specifically suppresses or degrades circRNA related to drug resistance, thereby restoring the activity of miRNA or regulating the relevant signaling pathways to enhance the efficacy of chemotherapy drugs [73]. Small molecule compounds or antibody drugs can also be developed to target the interaction between circRNA and proteins, thereby blocking the mediated drug resistance signaling [26]. Another strategy is combination therapy. Combining chemotherapy drugs with treatments targeting circRNA can improve treatment outcomes. For example, using circRNA targeted drugs or miRNA mimics during chemotherapy synergistically acts on cancer cells to overcome drug resistance. Combining chemotherapy with immunotherapy, targeted therapy, and other treatment modalities can also be considered to attack cancer cells from multiple levels and improve treatment effectiveness [78]. Personalized treatment is also an important approach to overcoming drug resistance. Owing to differences in circRNA expression patterns and drug resistance mechanisms among different patients, individualized treatment plans can be formulated by performing genetic testing and molecular typing to understand the individual circRNA characteristics and drug resistance status [24]. For patients with abnormal circRNA expression, corresponding targeted therapy drugs can be selected; for patients at high risk of drug resistance, more aggressive combination therapy plans can be adopted. Yi et al. found that circ_0007534 promotes paclitaxel resistance by sponging miR-625 and upregulating ZEB2, highlighting its role in chemoresistance pathways [18]. Fu et al. discussed how circPBX3 interacts with IGF2BP2 to stabilize ATP7A mRNA, contributing to platinum-based chemotherapy resistance [79]. In-depth exploration of the association between circRNA and chemotherapy resistance in endometrial cancer is beneficial for revealing drug resistance mechanisms, discovering new biomarkers, and providing a theoretical basis and practical guidance for developing effective strategies to overcome drug resistance [19, 80].

## Targeted therapy: a new perspective

### Target exploration for treatment

In the field of treatment for endometrial cancer, exploring effective therapeutic targets has always been a hot research topic. Because of its particular biological properties and significant association with the generation of endometrial diseases, circRNA has opened a new path for the investigation of targeted therapy targets [24]. CircRNA acts as a miRNA sponge involved in the biological regulation of endometrial cancer, making it a potential therapeutic target. Circ-ITCH, for instance, competes with miRNA-17 and miRNA-224 to bind to them, which causes its target genes, p21 and PTEN, to express differently [81]. When the PTEN gene mutates or loses activity, the phosphatase is inactivated, resulting in a loss of inhibition of cell proliferation, leading to the malignant transformation of cells and the induction of endometrial cancer. Therefore, it is possible to increase the expression of p21 and PTEN and prevent the growth and malignant transformation of tumor cells by focusing on the regulatory mechanism of circ-ITCH and developing medications or therapies that can improve its performance. RBPs can interact with some circRNAs as well. Circ-Foxo3, for instance, can bind to many proteins that interact with p21 and CDK2, inhibiting the cell cycle and halting the G1 to S phase transition [26]. Based on this, an in-depth exploration of the interaction network between circRNA and RBP, searching for key binding sites and regulatory pathways, may open new possibilities for searching for new targets for therapeutic treatment. By designing small molecule compounds or biological agents to specifically interfere with the binding of circRNA to RBPs, the cell cycle process can be regulated, and the growth of tumor cells can be inhibited [24]. In addition, circRNA can serve as a tumor marker for endometrial cancer, providing clues for target exploration. For example, hsa_circ_0109046 and has_circ_0002577 are upregulated more than twofold in the serum of endometrial cancer patients compared with healthy people [82]. Numerous signaling pathways and biological targets may be involved in the underlying regulatory processes of these very highly expressed circRNAs, which may be intimately linked to the genesis and progression of tumors. Comprehensive research on the roles and modes of action of these highly expressed circRNAs might result in the identification of novel therapeutic targets and serve as the foundation for targeted treatment.

Recent breakthroughs in the area of targeted therapy have placed a particular emphasis on the promise of circRNAs as precision medicine in conjunction with nanotechnology and other novel delivery methods. Such innovative approaches are intended to take advantage of the stability, specificity, and non-coding regulatory properties of circRNAs to create more precise therapeutic interventions in a range of disease models, particularly in cancer. As an excellent example, lipid nanoparticles (LNPs) can be used to deliver siRNAs against oncogenic circRNAs. A study has proposed aptamer-conjugated LNPs to deliver si-circPDHK1, which notably slowed down the growth and metastasis of clear cell renal cell carcinoma [83]. One more strategy used nanoparticles to silence circROBO1 and regulate the miR-130a-5p/CCNT2 axis to inhibit the progression of hepatocellular carcinoma [84].

Photothermal therapy is another area where circRNAs have been studied. As an example, Pan et al. [85] have discovered circBNC2 as a regulator of ferroptosis and made a nanoplatform co-delivering circRNA modulators and docetaxel to treat metastatic prostate cancer [85]. At the larger scale, a recent review by Racca et al. [86] highlights the various nanoparticle delivery approaches to circRNA-based antitumor therapies, further noting the convergence of RNA engineering and intelligent nanocarriers [86]. Regarding immunotherapy, circRNA and piRNA have been demonstrated to productively interact to promote PD-1/PD-L1 checkpoint blockade in cancer models, presenting a multi-modal immune evasion strategy [87].

### Combined therapy regimen

Owing to the complexity and heterogeneity of endometrial cancer, a single treatment method often fails to achieve the desired therapeutic effect [88, 89]. Therefore, the focus of research has shifted to combination therapies. The combination of circRNA targeted therapy with traditional treatment methods holds promise in enhancing treatment efficacy and reducing adverse reactions [90]. In terms of surgical treatment, the pre- or post- operative application of circRNA targeted therapy can help eliminate residual tumor cells and reduce the risk of recurrence. For example, in some locally advanced endometrial cancer patients, after tumor resection, the combined use of circRNA-targeted drugs specific to certain circRNAs can inhibit tumor cell proliferation and metastasis, thereby improving patient survival rates [91, 92]. CircRNA targeted therapy can increase tumor cells' susceptibility to chemotherapy and radiation during treatment. Research has indicated that certain circRNAs have the ability to control tumor cell death pathways and DNA damage repair systems. By inhibiting the expression or function of these circRNAs, tumor cells become more susceptible to the damage caused by radiotherapy and chemotherapy, thereby improving treatment outcomes. Furthermore, circRNA targeted therapy can also be combined with endocrine therapy. The occurrence of endometrial cancer is closely related to hormone levels such as estrogen, and endocrine therapy is a commonly used treatment method [93, 94]. The effectiveness of endocrine treatment can be improved by controlling the production of circRNAs linked to hormone signaling pathways. The estrogen receptor (ER), for instance, may be regulated by certain circRNAs. By targeting specific circRNAs, ER expression and activity can be altered, improving the sensitivity of endocrine treatment [24]. Additionally, the use of immunotherapy in cancer treatment is becoming increasingly widespread. The combination of circRNA targeted therapy with immunotherapy also shows great potential. CircRNAs can regulate the expression of immune cells and factors in the tumor microenvironment, affecting the immune evasion of tumors. By targeting specific circRNAs, the body’s anti-tumor immune response can be enhanced, thereby improving the effectiveness of immunotherapy.

### Current situation of clinical trials

Currently, clinical trials targeting circRNA for the treatment of endometrial cancer are still in the exploratory stage, but some preliminary results have been obtained. Some research teams are advancing clinical trials of targeted drugs against specific circRNAs. These drugs mainly work by inhibiting the expression or function of circRNA to achieve the goal of treating endometrial cancer. For example, through developing small interfering RNA drugs, relevant circRNA expression profiles associated with the occurrence of endometrial cancer can be intervened [18]. In preliminary in vitro and in vivo experiments, these RNAi drugs have shown certain anti-tumor activities. However, these clinical trials are still facing some challenges, such as drug delivery efficiency, target specificity, and safety [95]. In terms of combination therapy, some clinical trials are ongoing. For example, combining circRNA-targeted therapy with chemotherapy drugs to observe the efficacy and safety in endometrial cancer patients. According to preliminary findings, combination therapy may improve chemotherapeutic medication sensitivity while lowering dosage and side effects, offering a fresh approach to the treatment of endometrial cancer [96]. Some clinical trials are also exploring the use of circRNA as a tumor marker for the diagnosis and prognosis assessment of endometrial cancer. By detecting the expression levels of circRNA in a patient’s blood or tissues, the treatment response and prognosis status of patients can be predicted. These studies provide crucial evidence for the clinical application of circRNA in endometrial cancer. In conclusion, although clinical trials of circRNA-targeted therapy for endometrial cancer still face many challenges, with the continuous advancement of technology and deeper research, there is hope for new breakthroughs in the treatment of endometrial cancer.

### Exosomal circRNAs in intercellular crosstalk and therapy

CircRNAs found in exosomes are important in transferring signals between cancerous cells and may help with diagnostics and targeted drug delivery. By being encapsulated in extracellular vesicles, such as exosomes, they can interact with other cells, form the TME, support metastasis, and affect how cancer cells respond to therapy. Exosomes help transport circRNAs from one cell to another. Exosomal sorting relies on RNA-binding proteins and specific processes, which select some circRNAs as exosome cargo. The mechanism allows circRNAs to affect how genes are regulated, how cells look, and how they communicate with other cells. Zhang et al. focus on the molecules that control how circRNAs are encapsulated in exosomes and delivered, suggesting a role for these circRNAs in cancer progression [97].

Exosomal circRNAs are major players in cancer progression since they direct metastasis, help change the tumor environment, and promote resistance to chemotherapy. Such vesicle protected RNAs in exosomes regulate some processes like EMT, development of new blood vessels, and cell movement, increasing chances for cancer metastasis; in this respect, Li et al. discovered that exosomal circRNAs play a key role in making the tumor environment more favorable for the cancer to spread [98]. Exosomes not only lead to spread of cancer, but they also help to transform immune cells, fibroblasts, and endothelial cells so that they promote tumor development. According to Panda et al., exosomes help by stimulating signals from certain proteins to prepare the environment so that tumors can develop [99]. In addition, circRNAs found in exosomes play a role in chemo-resistance by influencing the work of drug transporters, apoptosis, and DNA repair steps. Wei et al. suggested in triple-negative breast cancer that circRNAs inside exosomes increase chemo-resistance, revealing their role as useful tool in treating the disease [100].

Exosomal circRNAs can be reliable non-invasive biomarkers, as they are stable in circulation and can be identified by their unique patterns in diseases. They are easily removed from blood and tested for diagnostic, prognosis, or treatment outcome purposes. Mukerjee et al. point out that exosomes are important for oncology monitoring, since circRNAs act as a reliable sign of tumor activity [101]. Similarly, Zhou and colleagues report that circRNAs found in exosomes are related to drug resistance and immune cell interactions, improving their suitability for treating diseases [102]. The exosomal platform also holds promise for therapeutic delivery. Engineered exosomes and synthetic nanocarriers can be loaded with therapeutic circRNAs or RNA inhibitors for targeted treatment. These delivery systems bypass biological barriers, achieve tissue specificity, and minimize immune reactions. Xiao et al. review how exosomes serve as intelligent carriers in lung cancer therapy, functioning both diagnostically and therapeutically [103]. Furthermore, Liu et al. highlighted that tumor-derived exosomes naturally home to tumor sites, enabling efficient RNA-based delivery [104].

## Diagnostic value assessment

### Early diagnosis application

Early diagnosis is extremely crucial for the treatment and prognosis of endometrial cancer. CircRNA has significant application value in the early diagnosis of endometrial cancer [24]. Detection based on CircRNA can serve as an auxiliary diagnostic method to improve the accuracy of early diagnosis of endometrial cancer. As mentioned earlier, there are differential expressions of circRNA in endometrial cancer tissues and normal tissues [25]. By detecting the expression levels of specific circRNAs, doctors can more accurately determine whether patients are suffering from endometrial cancer. For example, studies have found that in the serum of endometrial cancer patients, hsa_circ_0109046 and hsa_circ_0002577 show more than a twofold higher expression [105], indicating that detecting the expression levels of these CircRNAs in the serum may detect abnormalities in the early stages of the disease, providing clues for early diagnosis. The detection of CircRNA can also be used for the risk prediction of endometrial cancer. Some studies show that the expression levels of certain circRNAs are related to the risk of endometrial cancer. For example, in some high-risk groups, such as women with risk factors like obesity, hypertension, diabetes, etc., the detection of abnormal expression of specific circRNAs may indicate an increased risk of endometrial cancer [73]. Regular testing of CircRNA in these high-risk groups can help achieve early screening and prevention of endometrial cancer. CircRNA can also serve as a new type of molecular marker for monitoring the treatment efficacy and recurrence status of endometrial cancer. During the treatment process, assessing the changes in the expression levels of CircRNA can evaluate the effectiveness of the treatment [24]. If the expression levels of CircRNA return to normal after treatment, it may indicate a good treatment outcome; conversely, if the expression levels of CircRNA remain abnormal, it may suggest a risk of tumor recurrence or metastasis, requiring timely adjustment of the treatment plan [73]. However, to achieve the widespread use of CircRNA in the early diagnosis of endometrial cancer, some issues need to be addressed. For example, further optimization of detection methods is needed to enhance sensitivity and specificity; establishment of standardized detection procedures and reference value ranges is necessary to ensure the accuracy and comparability of test results; and large-scale clinical studies are required to validate the effectiveness and reliability of CircRNA as an early diagnostic marker [29].

### Current challenges in clinical translation

There are major obstacles in translating circRNAs from exosomes in the laboratory, in technology, and under regulations. Due to these barriers, the use of circRNAs as biomarkers for diagnostics and therapies remains limited in oncology and in the treatment of chronic diseases. It is often hard to study circRNAs because their levels vary significantly between individuals and sample types. CircRNAs are found in several biofluids (plasma, serum, urine), and their expression can change due to many reasons, such as the body’s clock, underlying diseases, and different ways of collecting and processing the samples. Because of these inconsistencies, it becomes tricky to decide how to detect certain diseases. For example, Zaporozhchenko et al. described differences in the amount of circulating cell-free RNA between individuals and over time, which can obscure important disease indicators [106]. In the same way, Chorley et al. believe that cohort studies with many people are necessary to determine standard levels of biofluid RNA [107].

Exosomal circRNAs are attracting attention because they remain stable and are selectively packaged inside the vesicles. Still, it is very challenging to isolate exosomes and increase the concentration of circRNAs. Common methods such as ultracentrifugation, size-exclusion chromatography, and immunoaffinity capture differ significantly in yield and purity. Different technologies make it harder to get the same results each time. The study points out that due to inconsistent ways of isolating exosomes, the results of circRNA quantification are not very reliable for use as clinical biomarkers [108]. Martinez-Dominguez et al. analyzed commercial and manual methods and found that they produced distinct results in both RNA yield and specificity [109].

Despite lots of scientific initiatives, having circRNA diagnostics accepted by regulators is still challenging. Unexpected results often occur because there are no set standards for handling samples, separating RNA, and making data comparisons standard. Absence of these makes it quite hard to meet the criteria that regulatory agencies such as the FDA and EMA require. According to the authors, having uniformity in the pre-analytical steps is important because even small differences may affect the final result and prevent biomarkers from being validated [110]. Besides, clear regulations to approve RNA-based liquid biopsies, especially those involving circular RNA, are still missing. Zhong et al. note that academic discoveries often fail to be used clinically since there is not enough strong data from large, prospective studies [111]. Additionally, Lawrence and Shah State that today’s regulatory frameworks do not fit the needs of circRNAs, which likely require flexible validation efforts [112].

## Future research directions

### Technical innovation needs

**Fig. 5:**
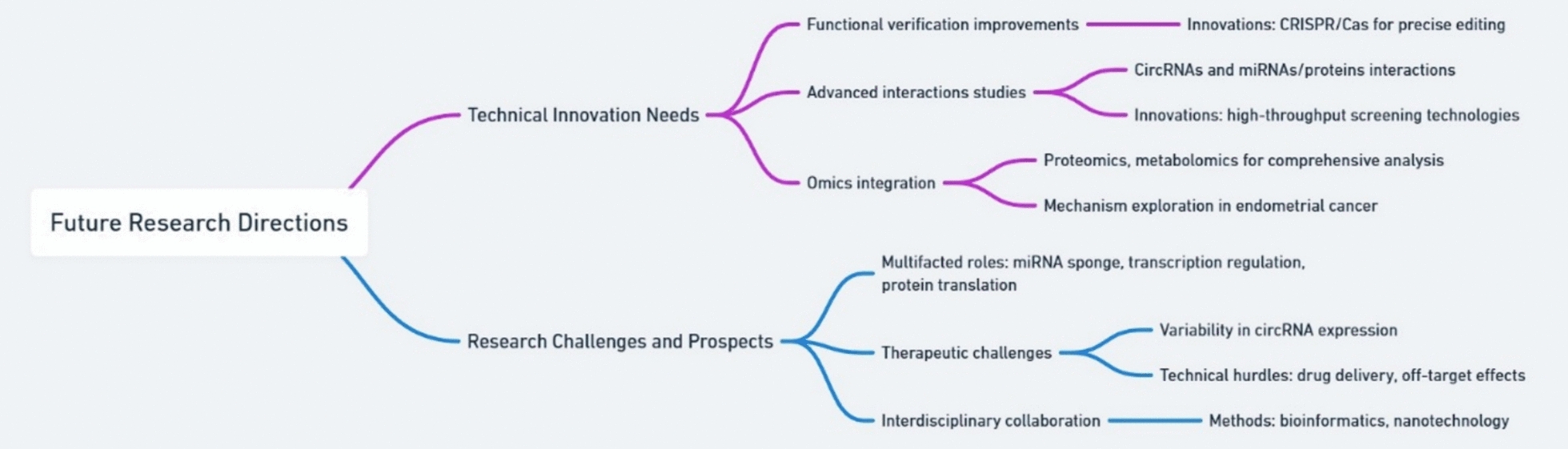
Mind map illustrating the future research directions for circRNAs in endometrial cancer

In the research field of circRNAs and endometrial cancer, technological innovation is a key factor in promoting deep research development (Fig. 5). Currently, despite some achievements, there are still numerous technical challenges that urgently require continuous exploration and innovation [24]. In terms of the detection technology of circRNAs, further improvement is needed. Existing detection methods, such as RNA sequencing, quantitative polymerase chain reaction (qPCR), etc., can detect the presence and expression levels of circRNAs, but they have certain limitations. For example, RNA sequencing is costly, and data analysis is complex; qPCR requires the design of primers specific to particular circRNAs, and its specificity and sensitivity need to be improved [22]. Therefore, it is necessary to develop more efficient, sensitive, and highly specific detection techniques to more accurately detect the expression of circRNAs in endometrial cancer tissues and cells. For instance, developing novel biosensors that can real-time and in situ detect changes in CircRNA expression provide stronger support for exploring its role in the occurrence and development of endometrial cancer [113]. Functional verification techniques of circRNAs also need continuous innovation. Currently, the main method of studying the function of circRNAs is through overexpression or knockdown to observe their effects on cellular biological behaviors. However, this method has certain limitations, such as potential off-target effects that impact the accuracy of experimental results [95]. Therefore, more precise and efficient functional verification techniques need to be developed. For example, using gene editing techniques like the CRISPR/Cas system to edit circRNAs at specific locations can provide a more accurate study of their function. Li et al. showed that RfxCas13d, paired with BSJ (back-splice junction) gRNAs, can effectively screen and validate functional circRNAs, demonstrating its potential in dissecting circRNA roles at the transcript level [114]. A follow-up by the same group expanded this tool to systematically identify regulatory circRNAs in cell lines, supporting its scalability [115]. In addition, integrating proteomics, metabolomics, and other omics technologies can comprehensively investigate the impact of CircRNAs on cell metabolism, signaling pathways, etc., and reveal their mechanism of action in endometrial cancer [24]. For example, to discover the novel ceRNA network in colorectal cancer, Chalbatani and team employed an integrative multi-omics approach [116].

Exploring the interactions between circRNAs and other molecules also requires new technical methods. CircRNAs can exert their biological functions by adsorbing miRNAs, binding proteins, etc., so in-depth research on the interactions between circRNAs and miRNAs, proteins, and other molecules is crucial for revealing their mechanism of action in endometrial cancer [29]. Currently, common research methods such as RNA immunoprecipitation (RIP), luciferase reporter gene assays, etc., although able to study their interactions to a certain extent, still have some limitations. For example, RIP experiments may be affected by antibody specificity; luciferase reporter gene assays may not reflect the real interactions in vivo [73]. Therefore, more advanced technologies need to be developed, such as high-throughput screening techniques based on protein-nucleic acid interactions, to more comprehensively and accurately study the interactions between CircRNAs and other molecules.

## Research priorities

To amplify the therapeutic potential of circRNAs, current efforts should focus on three essential aspects: efficient delivery systems, validation in large cohorts, and incorporation of artificial intelligence (AI) and bioinformatics. Although circRNAs are naturally stable and hold potential as therapeutics, their clinical use necessitate efficient delivery to a cell or tissue of interest. Progress in synthetic circRNA constructs has shown therapeutic promise, particularly in oncology and viral infections. There are, however, still difficulties in the way of biocompatibility, target specificity, and low immunogenicity. Cai et al. give an in-depth account of the latest developments in the field of synthetic circRNA therapeutics, such as chemical modifications or nanoparticle-based delivery [117]. Caporali et al. also note the potential of circRNA-based interventions in cardiovascular disease, but say that delivery is a technical bottleneck [118].

The majority of existing circRNA studies rely on small, frequently heterogenous patient groups, which restricts their translational value. The diagnostic and prognostic value of circRNAs need to be determined in large-scale and multicenter validation. Alqahtani et al. conclude that the potential implementation of personalized approaches to cancer therapy by combining pharmacogenomic data with circRNA profiling is promising, nonetheless, only on the condition that such associations are supported by well-powered population-level studies [119]. Likewise, Saleem et al. advocate for the systematic review and meta-analyse to assess the circRNA reproducibility in diverse biological systems [120].

The sheer amount of circRNA sequencing data and its structural complexity require advanced computational methods to be meaningfully interpreted. AI models, especially deep learning and graph-based methods, are being used more to recognize disease-specific circRNA signatures and to forecast circRNA–miRNA interactions. The article by Wang et al. specifies the use of machine learning to propel biomarker discovery in the analysis of non-coding RNA, which provides better sensitivity and specificity [121]. In addition, Aswathy et al. show how AI-powered models can be applied to miRNA studies and that this paradigm can be easily applied to circRNA [122]. As aided by these computational tools, multi-omics integration is getting to the center of the future of circRNA biomarker studies.

## Research challenges and prospects

The study on circRNAs and endometrial cancer has made certain achievements. However, there are still many challenges that need to be addressed urgently. Delving deeper into these challenges and actively seeking solutions is crucial for promoting research development in this field. CircRNAs have a multifaceted and intricate mode of action that is still unclear. Through a variety of mechanisms, including acting as miRNA sponges, controlling gene transcription, and taking part in protein translation, they can contribute to the onset and progression of endometrial cancer. However, there are still many unknowns about the specific mechanism of action of circRNAs in endometrial cancer. For instance, it is unclear how circRNAs preferentially bind to particular miRNAs and the regulatory mechanism behind this binding, despite the fact that some circRNAs have been shown to control the expression of target genes by absorbing miRNAs. Although further study is needed to determine the precise patterns of interactions and regulatory processes, CircRNAs may also influence the biological behaviour of cells through their interactions with proteins. Thus, more research into the mechanism of action of circRNAs is required in the future in order to uncover the intricate regulatory network behind their role in the onset and progression of endometrial cancer. There are still several obstacles in the way of the therapeutic use of circRNAs in endometrial cancer. To use circRNAs in clinical practice, a number of issues must be resolved, notwithstanding their potential as tumor indicators and therapeutic targets. For example, the expression of circRNAs varies among people and tissues, and the identification of universally significant circRNA indicators needs immediate attention. Targeted therapy with circRNAs also faces technical challenges, such as drug delivery and off-target effects. Currently, there is no effective method to specifically deliver CircRNA-targeted drugs to tumor cells, and how to avoid the impact of drugs on normal cells is also a problem that needs to be solved. Therefore, in the future, it is necessary to strengthen the clinical translation research of circRNAs and explore their application value in the diagnosis, treatment, and prognosis assessment of endometrial cancer. Interdisciplinary collaboration is also an important challenge and direction for future research. The study of CircRNAs and endometrial cancer involves multiple disciplines, such as biology, medicine, chemistry, physics, etc., and requires experts from various disciplines to collaborate and integrate interdisciplinary research methods and techniques in order to comprehensively and deeply study the mechanism of action and application value of CircRNAs in endometrial cancer. For example, combining bioinformatics and experimental biology methods to analyze and validate the expression data of circRNAs, utilizing the achievements of nanotechnology and materials science to develop efficient circRNA-targeted drug delivery systems, etc. Through interdisciplinary collaboration, it is hoped that new breakthroughs and progress can be made in the research on circRNAs and endometrial cancer.

## Data Availability

No datasets were generated or analysed during the current study.

## Abbreviations

ASO: Antisense oligonucleotides
circRNAs: Circular RNAs
circRNPs: CircRNA-protein complexes
EMT: Epithelial-mesenchymal transition
ESR1: Estrogen receptor 1
HDV: Hepatitis D virus
HRT: Hormone replacement therapy
ITCH: Itchy E3 ubiquitin ligase
MREs: MicroRNA response elements
ncRNAs: Noncoding RNAs
PCOS: Polycystic ovary syndrome
qPCR: Quantitative polymerase chain reaction
qRT-PCR: Quantitative reverse transcription polymerase chain reaction
RBPs: RNA binding proteins
RIP: RNA immunoprecipitation
